# Contribution of insect gut microbiota and their associated enzymes in insect physiology and biodegradation of pesticides

**DOI:** 10.3389/fmicb.2022.979383

**Published:** 2022-09-14

**Authors:** Saleem Jaffar, Sajjad Ahmad, Yongyue Lu

**Affiliations:** ^1^Department of Entomology, South China Agricultural University, Guangzhou, China; ^2^Key Laboratory of Integrated Pest Management of Crop in South China, Ministry of Agriculture and Rural Affairs, South China Agricultural University, Guangzhou, China; ^3^Key Laboratory of Natural Pesticide and Chemical Biology, Ministry of Education, South China Agricultural University, Guangzhou, China

**Keywords:** symbiotic microbes, enzymes, pesticides, non-target organisms, metabolic pathways

## Abstract

Synthetic pesticides are extensively and injudiciously applied to control agriculture and household pests worldwide. Due to their high use, their toxic residues have enormously increased in the agroecosystem in the past several years. They have caused many severe threats to non-target organisms, including humans. Therefore, the complete removal of toxic compounds is gaining wide attention to protect the ecosystem and the diversity of living organisms. Several methods, such as physical, chemical and biological, are applied to degrade compounds, but as compared to other methods, biological methods are considered more efficient, fast, eco-friendly and less expensive. In particular, employing microbial species and their purified enzymes makes the degradation of toxic pollutants more accessible and converts them into non-toxic products by several metabolic pathways. The digestive tract of insects is usually known as a superior organ that provides a nutrient-rich environment to hundreds of microbial species that perform a pivotal role in various physiological and ecological functions. There is a direct relationship between pesticides and insect pests: pesticides reduce the growth of insect species and alter the phyla located in the gut microbiome. In comparison, the insect gut microbiota tries to degrade toxic compounds by changing their toxicity, increasing the production and regulation of a diverse range of enzymes. These enzymes breakdown into their derivatives, and microbial species utilize them as a sole source of carbon, sulfur and energy. The resistance of pesticides (carbamates, pyrethroids, organophosphates, organochlorines, and neonicotinoids) in insect species is developed by metabolic mechanisms, regulation of enzymes and the expression of various microbial detoxifying genes in insect guts. This review summarizes the toxic effects of agrochemicals on humans, animals, birds and beneficial arthropods. It explores the preferential role of insect gut microbial species in the degradation process and the resistance mechanism of several pesticides in insect species. Additionally, various metabolic pathways have been systematically discussed to better understand the degradation of xenobiotics by insect gut microbial species.

## Introduction

In modern agriculture, for the management of various kinds of pests and the production of high-yield crops to meet the food availability for human beings, pesticides are extensively applied all over the world (Giambò et al., 2021). Pesticides are chemicals that control different pests such as rodents, arthropods, weeds and microbial pathogens (Huang and Chen, 2022). Pest management strategy is a vigorous arms race: on the one hand, farmers, pesticide inventors, agribusiness men, and researchers throughout the world struggle for the protection of crops and their higher production (Damalas and Koutroubas, 2018). While on the other hand, insects and other microbial pathogens follow their biological metabolism and drive to live and reproduce their generations (Pietri and Liang, 2018). Due to the repetitive application of pesticides with higher concentrations, insects and other pathogens fail to control them and develop cross-resistance (Daisley et al., 2018; Gressel, 2018). However, insect resistance against insecticides produces severely threaten non-target living organisms and contaminates the ecosystem (Khalid et al., 2021). Various studies have reported that pesticides’ toxic residues are abundantly present in soil, sediments, and water bodies (Mulla, Ameen et al., 2020).

These hazardous compounds and their toxic metabolite residues significantly affect the climate and living organisms such as soil biota, fish, birds, mammals, plants and human beings (Lee et al., 2021; Pujar et al., 2022). In addition, their toxic residues ruin organisms’ behavior, reproduction cycles and metabolism mechanisms, which can permanently alter the interrelated ecosystem (Zhao et al., 2019). These toxic compounds are degraded into simpler or less toxic substances using various methods such as chemical reactions, physical methods, photodegradation and biodegradation. Compared to other techniques, biological methods are less expensive, environment-friendly, more effective and easier to adapt to remove emerging pollutants (Hao et al., 2018).

Microbial species have been extensively applied for the biodegradation of environmental pollutants, including agrochemicals (Chen et al., 2015). To date, researchers throughout the world have screened millions of microbial species (bacteria, fungi, yeasts, algae, etc.) from the soil, sewage sludge, wastewater and other contaminated sites (Yang L. et al., 2021). Investigation of pure cultures of microbial species has revealed that toxic molecules are transformed into various metabolites (Ahlawat et al., 2020). Nevertheless, due to considering prominent features of insect gut microbial species like high resistance to pesticides and purification of novel suitable enzymes, various researchers have isolated a diverse number of microbial species for the biological treatment of wastewater and clean-up of the contaminated environment (Skidmore and Hansen, 2017).

**FIGURE 1:**
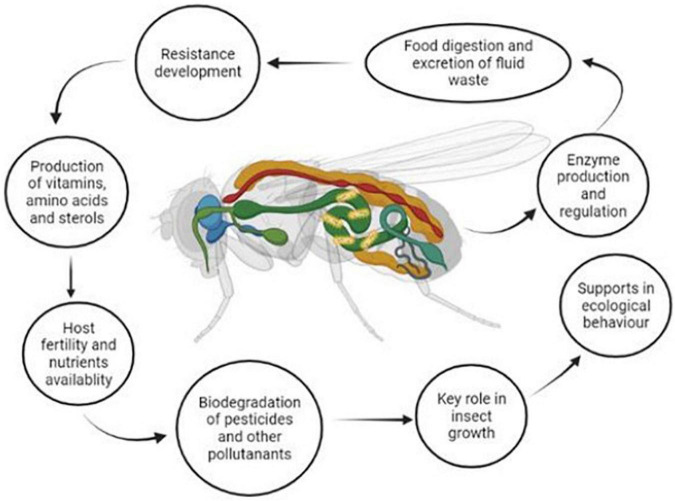
Role of insect gut microbiota in insect physiology.

Gut microbial species play a pivotal role in the detoxification, mineralization, and catabolism of organic molecules employed in pest control as determined by degradation or histochemical mechanisms (Berasategui et al., 2016). They are also considered superior organs for producing pheromones, synthesizing vitamins, and different enzymes to prevent pathogens (Ozdal et al., 2016). The gut of insects and other arthropods provides a rich nutrient medium for developing microbial species that can produce some essential enzymes and contribute significantly to insect physiology (Ramakrishnan et al., 2019; Figure 1). Insect gut microflora provides a prominent environment for transforming genes, mutant traits, and conjugative plasmids, which can adapt to harsh environmental conditions and perform smoothly in biodegradation processes (Xia et al., 2018).

More importantly, microbial species isolated from this source are rarely indigenous to the polluted environment. Hence, their use in bioaugmentation and biodegradation enhances their efficiency to remove environmental pollutants (Kadri et al., 2018). To keep in mind these critical points, insect associated-microbial species, especially bacteria, are more vigorous and beneficial because they are interrelated with the application of active ingredients (Blanton and Peterson, 2020). The coordination between symbiotic microbial species and resistance to pesticides in arthropods would provide new opportunities for managing pests and isolating efficient microbial species to protect the agroecosystem (Li et al., 2018).

This review investigates the resistance mechanisms of different pesticides in insect pathogens and the transforming mechanisms of their parent toxic compounds into less toxic intermediates by the isolation of gut microflora. Additionally, microbial species interlinked with insects and involved in the detoxification of pesticides will be essential in the designing of future novel ingredients to ensure their long-term efficiency. Therefore, investigating the linkages between environmental contaminants and gut microflora is of great significance. This literature review will be beneficial and guider to reveal the possible impacts of gut microflora on the fate of organic pollutants and provide a more comprehensive insight into the mineralization, transformation, and biodegradation of pesticides and other emerging pollutants from the environment.

## Fate of pesticides in the environment

Throughout the world, an extensive range of pesticides like insecticides, herbicides, rodenticides, fungicides, nematicides, molluscicides, rodenticides, bactericides, repellents, insect growth regulators and disinfectants have been generated for the management of specific target pests in agriculture, aquaculture, horticulture and households (van de Merwe et al., 2018). Currently, more than 3.5 million tons of pesticides are used throughout the world, out of which 47.5% are herbicides, 29.5% are insecticides, 17.5% are fungicides, and 5.5% are other types of pesticides (Sharma et al., 2019). Since its inception 50 years ago, China has grown to be the world’s largest producer and consumer of pesticides. The other major pesticide-consuming countries are the United States, Argentina, Thailand, Brazil, Italy, France, Canada, Japan, and India (Olisah et al., 2019). These agrochemicals are extensively introduced into modern agriculture and urban ecosystems during their production, transportation, improper storage and unwise applications, which cause severe environmental threats (Ahmad et al., 2021). Local governments and environmental protection agencies regulate the production of pesticides and their applications. But the ecological management and risk assessment rules and regulations are generally restricted to formulating agrochemicals and their active ingredients and additives (Pokhrel et al., 2018). According to a combined statement of WHO and UNEP, approximately 200,000 people worldwide die, and roughly three million are affected yearly by pesticide residues (Meftaul et al., 2020). Another study revealed that the majority of cases, nearly 95% of them are reported from developing countries (Yadav et al., 2015). Agrochemicals severely effect the ecosystem through toxic residues at the application sites, such as agricultural farms, lawns and parks (Wu et al., 2007). However, these compounds pose severe threats to aquatic organisms by leaching down into the groundwater and through surface runoff into lakes, rivers, and other water bodies (Ahmad et al., 2022a). Furthermore, when pesticides are applied to crops, horticulture areas, home lawns and school parks, many people including, children and women, animals, beneficial arthropods, birds and wildlife creatures, are seriously affected (Masud et al., 2018).

Due to unwise applications of pesticides with higher concentrations, their toxic residues have been frequently revealed in the urban air, dust, soil and water bodies than in those of rural areas, predominantly due to primary, secondary and re-emissions of the parent compound and their toxic derivatives (Ren et al., 2018). Agronomic crops and other ornamental plants can easily absorb these chemicals from contaminated sites and transfer them to their vegetative and reproductive parts (Kim et al., 2017). When farmers apply higher concentrations of pesticides to protect their crops from pests and diseases, their residues are entered into food commodities (Dai et al., 2010). However, various researchers are working to investigate toxic pesticide residues in fruits and vegetables growing in agricultural, rural and urban areas in developing and developed countries (Pietrzak et al., 2020; Barbieri et al., 2021; Zamule et al., 2021). Incorporating pesticide residues into daily food consumption is a foremost safety issue for consumers worldwide (Hasan et al., 2017). The excessive use of pesticides deliberately affects flora, fauna and the ecosystem (Arunkumar et al., 2017). We briefly discuss the risk of pesticides to humans’ health and other non-target living organisms in the following sections.

### Human health

The labors working in pesticide formulation industries, agriculture areas, and assassinators for managing household pests are generally affected by direct or indirect pesticide exposure. There are higher chances of risk for people working in the pesticide manufacturing industries at the time of formulation, packaging and production because they handle crude materials and other hazardous solvents (Gangemi et al., 2016; Nicolopoulou-Stamati et al., 2016). Various kinds of health disorders such as cancer, diabetes problems, respiratory issues, neurological disorders, reproductive syndromes and oxidative stress are produced due to direct or indirect exposure and handling of pesticides or their toxic active ingredients in foodstuffs (Carles et al., 2017; Grewal, 2017; Rani et al., 2021). Some studies have revealed that due to continuous risk assessment of highly toxic compounds, including pesticides such as lung cancer, breast cancer, leukemia and multiple myeloma have occurred in human beings (Han et al., 2018; Ruiz et al., 2018; Huang et al., 2019; Jaacks et al., 2019). Meng et al. (2016) carried out a study to investigate agrochemical exposure in indoor dust and blood samples. Results of this study revealed asthma is positively interlinked with exposure to alpha-hexachlorocyclohexane in humans.

In another study to evaluate pesticide exposure and its effects on human health, Tanner et al. (2009) carried out a study. They discovered that Parkinson’s disease and 2-4 D herbicides are closely associated with their cause. Yan et al. (2016) reported that pesticides and Alzheimer’s disease are closely interlinked, and a meta-analysis proved that pesticide exposure is hazardous for the brain and eyes. Recently, Shah et al. (2020) carried out a study investigating the effect of organochlorine pesticides such as β-hexachlorocyclohexane, dichlorodiphenyldichloroethylene and dieldrin on human epithelial ovary cells for the risk prediction of ovarian cancer. The findings of this study revealed that organochlorine pesticides highly affect human health and stimulate the measurement of reactive oxygen species (ROS), pro-inflammatory response and DNA damage in human epithelial ovary cells. Besides this, DDT organochlorine pesticide caused DNA damage, genetic instability, micronucleus formation and the sister chromatid exchange in humans (Yáñez et al., 2004; Savant et al., 2018). Cassidy et al. (2005) studied the relationship between women’s breast cancer and heptachlor pesticide. The results of this study revealed that women’s breast cancer risk was positively correlated with the level of heptachlor epoxide. Richardson et al. (2014) investigated the effect of dichlorodiphenyltrichloroethane (DDT) and dichlorodiphenyldichloroethylene (DDE) on human health and found that these both pesticides were responsible for causing Alzheimer’s disease. In another study, due to high exposure to organochlorine pesticides their toxic effects on human health were examined, and it was found that these pesticides cause Parkinson’s disease (Freire and Koifman, 2012).

### Impacts on water bodies

Imprudent application of pesticides in farming could pollute surface water *via* draining, runoff, leaching and drift. Polluted surface water harms non-target organisms, including humans and animals (Chrustek et al., 2018; Lee and Choi, 2020). Surface water is considered a major drinking water source in developing nations such as Pakistan, India, Bangladesh, Nepal, and Sri Lanka (Mojiri et al., 2020; Teklu et al., 2022). The residues of various pesticides from the groups of organochlorines, organophosphate, carbamates, neonicotinoids, and pyrethroids are found in the rivers of California (Anderson et al., 2018). Besides this, several other European countries also investigated pesticide residues and noticed that 76 types of pesticide residues are present in European soil. Furthermore, it was revealed that 83% of soil contained one type of residue, and 58% of soil contained two, three or more types of residues. The highest concentrations of glyphosate and its derivatives were detected frequently. The presence of pesticide residues in the surface water and rivers all over the world causes critical threats to aquatic organisms (Mitchell et al., 2017; Tian et al., 2018; Dias et al., 2020).

A study was conducted to evaluate the exposure of organochlorine and pyrethroid pesticide residues in surface water, fish, sediments and aquatic weeds in the southern region. Results of this study revealed that residues of organochlorine pesticides were identified in surface water, sediments, fish muscle, gills, liver, and aquatic weeds at a concentration of 0.001–34.44 μg/L, 0.01–16.72 μg/Kg, 0.01–26.05 μg/Kg, 0.01–40.56 μg/Kg, 0.01–65.14 μg/Kg, 0.01–5.53 μg/Kg, respectively. This study further explained that organochlorine pesticides such as eldrin, dieldrin, endosulfan, endrin, and heptachlor were the prominent pesticides identified with the above level of maximum residue limit set by the World Health Organization in surface water, sediments and fish (Arisekar et al., 2019). In another study, residues of pesticides in the Guayas River at 181 places were investigated using the solid phase extraction method. Results of this study explained that 26 types of pesticide residues in fresh water at 108 sampling sites (60%) were detected with higher concentrations. The major types of pesticides found in river water are cadusafos, butachlor, and pendimethalin at 62, 21, and 21, with concentrations of 0.081, 2.006, and 0.557 μg/L, respectively. Finally, this study also demonstrated that all detected pesticides in river water are frequently found in agriculture and horticulture crops such as rice and banana, with higher concentrations due to irregular application methods like aerial spraying. Finally, their residues are transferred to the rice field and river water. This study also suggested that precaution measures such as legal regulations and awareness campaigns for farmers and local industries are highly recommended to control environmental contamination and prevent the accumulation of pesticide residues in aquatic and terrestrial systems (Deknock et al., 2019).

Recently, for the investigation of highly used herbicide residues such as atrazine, acetochlor, alachlor, hexazinone, metolachlor, simazine, terbuthylazine, trifluralin, and phenoxy acids (MCPA and 2,4-D) in two type of fishes (*Clarias gariepinus*, *Oreochromis mossambicus*) an experimental study was conducted. This study showed that all herbicides’ residues were found in analyzed samples with a total concentration ranging from 42.3 to 238 ng/g in *Clarias gariepinus* and 72.2–291 ng/g in *Oreochromis mossambicus*. The most dominant herbicides which are found in fish tissues, gills and liver are phenoxy acid herbicides, acetochlor, atrazine and terbuthylazine with the ranges of 17.6 ± 12 ng/g, 28.9 ± 16 ng/g, 15.4 ± 5.8 ng/g, 12.7 ± 7.1 ng/g, 12.4 ± 12 ng/g, respectively (Tyohemba et al., 2021).

### Threats to beneficial arthropods

Insect pollinators and predators play a pivotal role in developing many crops, as insect pollinators increase the yield and predators protect crops from pest infestation (Ihara et al., 2017). But due to excessive use of agrochemicals and unselective treatment in the modern agriculture system, their diversity and abundance are severely affected (Joshi et al., 2020). It has been reported that only 1% of pesticides reach the target site, whereas the remaining amount accumulates in the environment and contaminates it (Goergen et al., 2016; Fine et al., 2017; Stein et al., 2017). Beneficial arthropods are directly linked with pesticide exposure at the time of application or immediately after applying pesticides. The droplets of toxic residues could inlet on their cuticle by ingestion and influence their growth and mating behavior (Sgolastra et al., 2017).

Abraham et al. (2018) investigated the effects of glyphosate herbicides on beneficial insects (*Apis mellifera, Hypotrigona ruspolii*) under laboratory conditions. The bees were treated with the recommended concentration, a two-fold higher recommended concentration, and distilled water for control. The impact of glyphosate herbicide was compared with the lambda cyhalothrin. The herbicide was sprayed on plants as well as the bees were treated with herbicide-sprayed filter paper. Results of this study revealed that a more significant number of bees died after contact with the herbicide in both ways. This study concluded that spraying glyphosate herbicide was very dangerous for beneficial insects by contacting or spraying on fresh plants with more than the recommended dose. Another study investigated the effects of widely used neonicotinoids (acetamiprid, imidacloprid, thiamethoxam, and thiacloprid) on spiders. All the neonicotinoids with recommended doses were applied in field conditions, and short-term exposure was evaluated on spiders. Results of this study revealed that after 1 h, imidacloprid showed more critical effects and revealed partial acute lethality (15–32%). Acetamiprid showed strong sublethal effects, particularly when employed dorsally on *Philodromus cespitum*. After 1 day of application of thiacloprid and acetamiprid, *Linyphiidae* species were paralyzed or finally caused death, especially in males (Øezáè et al., 2019).

A study was conducted to reveal the toxicity of imidacloprid, thiamethoxam and sulfoxaflor on the aphid (*Aphis gossypii*). The impact of these pesticides on natural enemies of aphids, especially parasitoids (*Aphidius colemani*), was investigated with low lethal, median lethal and sublethal concentrations. This study showed that the median lethal concentration caused maximum mortality of parasitoids compared to sulfoxaflor, while imidacloprid had the least negligible impact on the diversity of parasitoids (Ricupero et al., 2020). To study the effects of more neonicotinoid pesticides such as imidacloprid, thiamethoxam, clothianidin and dinotefuran on the parasitoid larvae (*Coccinella septempunctata*) by the application of lethal and median lethal doses using the direct contact method. This study indicated that neonicotinoid pesticides are hazardous for the survival of larvae. The median lethal dose highly impacts the emergence of larval instars, pupal emergence and weight. Finally, this study concluded that pesticide application is hazardous for the survival of beneficial insects, primarily involved in integrated pest management services (Wu et al., 2021).

### Toxic effects on plants and animals

Every day, increasing environmental pollution affects many living organisms and has always been considered a critical challenge in the scientific community (Niroumand et al., 2016). The high accumulation of pesticides in agricultural soils and their cumulative behavior and toxicity pose severe threats to beneficial plants (Ferrando and Matamoros, 2020). It is well known that the accumulation of pesticides affects the behavior of soil microbial species and enzymes and is absorbed by plants, which further transfers it to non-target organisms through the food chain process (Sayed et al., 2020). The adaptation of medicinal plants to cure various diseases has been practiced for several centuries and even today plays a pivotal role in primary health care as a therapeutic agent in several developing nations (Reinholds et al., 2017; Kumar et al., 2018).

The application of herbal medicines to treat various illnesses has increased significantly in the past few decades due to their prominent features such as minimum side effects compared to synthetic drugs, inexpensive and excellent viability (Ammar et al., 2020). Besides their numerous benefits, toxic pesticide residues could be more dangerous and cause many diseases in humans and other living organisms (Righi et al., 2018). Recently, Luo et al. (2021) have studied the accumulation of pesticide residuals in various medicinal plants, which are frequently used throughout the world for the welfare of humanity. Results of this study explained that in 1771 samples, 88% of pesticide residues were detected. Terrifyingly, 59% of pesticide residues are beyond the European Pharmacopoeia (EP) limit and 43% are confined to 35 types of banned pesticides worldwide. Additionally, this study demonstrated that eight pesticide residues were five hundred times higher than the default maximum residue limit set by the environmental protection agency.

In another study, Li R. -X. et al. (2020) investigated the presence of various pesticide residues in herbal plants using the Quick Easy Cheap Effective Rugged Safe (QuEChERS) extraction method. All residues were detected through Ultra-Performance Liquid Chromatography and Gas Chromatography-Mass spectrometry analysis. This method was applied to 39 real samples of *Ophiopogon japonicus*, *Polygonatum odoratum*, and *Paeonia suffruticosa* obtained from different locations, and the results of this study revealed that in 92.3% of samples, residues of pesticides were detected. This study showed that 26% pesticide residues are frequently detected in three traditional Chinese medicine plants. In addition, tebuconazole and paclobutrazol residue levels were considerably higher in nine samples compared to the maximum residue limit.

The primary way of transformation of pesticides in the general population is the consumption of food commodities that might be polluted with toxic residues of pesticides (Nagy et al., 2020). However, their residues can ultimately be inserted into animals’ digestive tracts *via* various pathways and affect their physical conditions (Altun et al., 2017; Yuan et al., 2019). Recently, Nerozzi et al. (2020) investigated the effects of glyphosate and its most famous formulation Roundup, on animal health and reproductive functions. In this study, the pig was chosen as a model animal. The commercial semen of pigs was treated with glyphosate and Roundup formulation at 0–360 μg/mL concentrations and incubated at 38°C for 3 h. The consequences of this study indicated that the application of high concentrations of glyphosate significantly reduced sperm viability, motility, mitochondrial activity and acrosome integrity. While on the other side, by treating lower concentrations (5–100 μg/mL) of Roundup formulation, all the disorders were observed after 1 h of incubation. Finally, this study concluded that pesticides active ingredients and inert materials negatively affect animals and the human reproductive system. Jarrell et al. (2020) also reported that glyphosate-based herbicides are more dangerous for animals’ health and cause severe diseases such as the reproductive system, altering the regulation of enzymes, disrupting serum levels and activity, and loss of fertility.

An investigation was carried out to evaluate deltamethrin and ivermectin residues on local sheep milk and meat. A total of eighty samples (40 each for milk and meat) were obtained from different places, and detection of pesticide residues was observed by performing High-Performance Liquid Chromatography. Results of this study indicated that 92.5 of milk samples and 90% of meat samples were polluted with toxic deltamethrin residues. More alarmingly, this study highlighted that all samples were contaminated with ivermectin residues above the maximum residue limit set by the World Health Organization (WHO) and Food and Agriculture Organization (FAO) (Mani and Al Araji, 2022).

However, to remove various environmental pollutants, protect the diversity of living organisms, and save crops from pests, effective, eco-friendly, less expensive, and more applicable methods are urgently required.

## System biology-based approaches for the pesticides degradation in agroecosystem

In order to gain a better knowledge of plants and microbes, researchers are using system biology technologies (Bhatt et al., 2016). Numerous details on the interactions between microbes, plants, humans and other non-target organisms by pesticides in nature have been fabricated because of advances in the fields of genomics and proteomics (Bhandari et al., 2021). Recently, a biological system-based approach was carried out to remove atrazine residues from the contaminated environment and to understand the complex biological network with different cellular systems modeling and simulation of atrazine were performed. The findings of this study revealed that two functional enzymes from bacteria (chlorohydrolase and monooxygenase) actively performed and completely degraded atrazine from the environment. To learn more about the biochemistry and physiology of atrazine in various cellular networks, additional analysis and simulations of the utilized model were performed. Atrazine degradation’s 289 nodes and 300 edges were verified by topological analysis (Bhatt et al., 2020). Insecticides containing pyrethroids are frequently used to control pests in homes and agricultural crops (Bhatt et al., 2022b). The complete removal of various pyrethroid pesticides was achieved using a system biological based approach and a simulated model. Results of this study explain that the toxic metabolites of pyrethroids severely affect non-target organisms, especially beneficial arthropods, microorganisms and human health. In addition, this investigation actively contributed to analyzing the toxicity and removal of other emerging pollutants from the agroecosystem (Bhatt et al., 2021a). In another study, a potential bacterial strain, *Bacillus subtilis* 1D, was isolated from a polluted agriculture field and investigated their degradation efficiency to degrade cypermethrin from the environment. The findings of this study showed that bacterial stain efficiently degraded 95% of cypermethrin within 15 days and converted it into various metabolites. In addition, laccase and esterase enzymes were identified from bacterial strain and observed that both enzymes more rapidly degraded cypermethrin as compared to free cells (Gangola et al., 2018).

A biological molecular model and a purified methyl transferase enzyme were adopted to degrade residues of methyl halide from a polluted environment. This study explained that the enzymes played a crucial role in the remediation of methyl halide. In addition, this model demonstrates that a volatile poisonous substance impacts the earth’s environmental layers and life systems (Bhatt et al., 2019). A potential *Bacillus* sp. FA3 was isolated from the contaminated environment and examined their degradation efficiency using the Box-Behnken design to degrade fipronil from the soil and water systems. Results of this study revealed that at optimum conditions (temperature of 32°C, pH 7, and rotational speed of 110 rpm), the bacterial strain performed efficiently and degraded 76% of fipronil within 15 days. Finally, this study concluded that *Bacillus* sp. FA3 was a superior candidate for removing fipronil from the wastewater and soil system and could be helpful for large-scale treatment (Bhatt et al., 2021b).

## Diversification of symbiotic microbiota and their role in insect physiology

A diverse range of symbiotic microbial species have been produced within the insect gut and have contributed a very significant role in the regulation of insect metabolism, enhanced food digestion, increased excretion of waste fluids, protecting the host from enemies, developing resistance against toxins and degrading them into their intermediates (Smith et al., 2017; Heys et al., 2018; Tokuda et al., 2018; Hauffe and Barelli, 2019; Figure 1). The identification and characterization of insect gut microbial species are investigated mainly by culture-dependent or culture-independent techniques (Bourguignon et al., 2018; Bruno et al., 2019). However, the culture-dependent method usually produces biased results. It relies on various parameters and techniques, while in the culture-independent method, a lot of omics and molecular approaches are applied, such as 16S rRNA and BLAST analysis, which provide a better and more comprehensive picture of the microbial communities located in insect guts (Eski et al., 2018; Erlandson et al., 2019; Erb and Kliebenstein, 2020). The application of high throughput and next-generation sequencing provides new insights into obtaining microbial ecology (Harishankar et al., 2013). It reveals that the diversity of microbial species by using independent culture methods identified a higher number of microbial communities than traditional culture-based and conventional molecular methods (Bhatt et al., 2021c,a, 2022a; Mishra et al., 2021; Ahmad et al., 2022a). Therefore, a comprehensive evaluation of microbial communities within a host species plays a vital role in understanding insect physiology and their interactions with insect hosts (Armitage et al., 2022).

A comprehensive investigation was carried out to evaluate insect symbiotic microbial species and their significant roles in 305 insect samples belonging to 218 insect species in 21 taxonomic orders. Using an independent culture method and adopting 16S rRNA analysis, 454 pyrosequencing were performed, and 174,374 sequence reads were gained. This study’s results indicated a total of 9301 bacterial operational taxonomic units (OTUs) at a distance level of 3% from all samples, with an average of 84.3% (± 97.7) OTUs per sample. In addition, this study suggested that gut microbial species were dominated by *Proteobacteria, Wolbachia*, and *Firmicutes* with a ratio of 62.1, 14.1, and 20.7%, respectively. Finally, this study concluded that these bacterial communities could help in food digestion, the development of larval stages and enhanced the insect immune system (Yun et al., 2014). The findings of another study showed that the hindgut of subterranean termites contained a 90% population of bacteria and archaea (Hongoh, 2010). The diversity of bacterial species in the digestive tract of fruit flies (*Drosophila melanogaster*) was studied using 454 pyrosequencing of 16S rRNA gene amplicons. Results of this investigation explained that 5 OTUs enriched the sequence reads, and ≤97% of that sequence identity could be related to *Acetobacter pomorum*, *Acetobacter tropicalis*, *Lactobacillus brevis*, *Lactobacillus fructivorans*, and *Lactobacillus plantarum* (Wong et al., 2011).

In another study, using an independent culture technique and adopting molecular approaches such as denaturing gradient gel electrophoreses and 16S rRNA analysis, a high diversity of genus *Gammaproteobacteria* were identified in the gut of the locust *Schistocerca gregaria.* The results of this study suggested that this diversity of bacterial species engaged with a defensive mechanism and enhanced it against external pathogens and toxic chemicals (Dillon et al., 2010). Recently, Xue et al. (2021) investigated the diversity of gut microbial species in various life stages of *Adelphocoris suturalis* by adopting the independent culture technique. Results of this study explained that the gut of the first and second instar was highly accomplished with the diversity of bacterial species. This study demonstrated that in the phylum, Proteobacteria and Firmicutes were dominant with a ratio of 87.06 and 9.43%, respectively, while at the genus level, *Erwinia* (28.98%), *Staphylococcus* (5.69%), and *Acinetobacter* (4.54%) were dominant bacteria. Finally, this study concluded that the diversity of bacterial species could be applied for biological control.

## Functions of insect gut microbiota

The insect gut is divided into three primary regions: the anterior midgut or foregut, the posterior midgut, and the hindgut (Wang G. -H. et al., 2020). The anterior midgut and hindgut arise from the embryonic epithelium. They are sheltered from pathogens by an exoskeleton of chitin and integument glycoproteins, while the posterior midgut is mainly used for absorption and digestion (He et al., 2018). Additionally, the hindgut of insects serves as an extension of the body cavity and is used to collect dietary waste (Siddiqui et al., 2022). However, it offers an appropriate environment that stimulates the proliferation and diversification of insect gut microbiomes (Bruno et al., 2019). Many studies have reported that insect gut microbiota plays a significant role in developing symbiotic insect interactions facilitated by secondary metabolites (Shang et al., 2021a). Besides this, they also play an essential role in the detoxification of pesticides, providing a natural defense system, nutrient availability, development of resistance against toxins and pathogens, breakdown of food, and suitable for proper growth of insects (Jang and Kikuchi, 2020; Jing et al., 2020; Mogren and Shikano, 2021; Tilottama et al., 2021).

High benefits and more prominent features of insect gut microbial species provide new insights into the development of beneficial arthropods, which are often used as biocontrol agents to solve environmental problems and further applications for the welfare of humans (Samoilova et al., 2016; Borrelli et al., 2017; Liao et al., 2017). However, considering the superior features of insect gut microbiota, this review mainly focuses on their potential applications for the detoxification of pesticides and their toxic metabolites for the cleanup of the environment (Wang S. et al., 2020).

### Development of resistance against pesticides

Pesticides have been applied to manage pests and diseases since the start of agriculture for the production and protection of crops. However, the unwise use of pesticides accumulates in the ecosystem and contaminates plants, air, water and soil (Lewis et al., 2016). The storage of pesticides in plants can develop resistance or tolerance against various pests (Ramakrishnan et al., 2019). A lot of studies have demonstrated that resistance is also developed due to reduction of toxicity of a compound, the introduction of a new pesticide group, target site mutation or over expression, pre-date or wrong selection of pesticide, repetition of the same chemical, environmental changes, and degradation of parent compounds into their metabolites by insect gut microbiota and their detoxifying enzymes (Naik et al., 2018; Hawkins et al., 2019; Matsuda et al., 2020; Table 1).

**TABLE 1:** Pesticide resistance cases in various insects mediated by gut microbial species.

Name of pesticide	Insect common name	Insect scientific name	Gut microbiota	References
Prothiofos	Diamondback moth	*Plutella xylostella*	*Pseudomonas* sp., *Stenotrophomonas* sp., *Acinetobacter* sp., and *Serratia marcescens.*	Indiragandhi et al., 2007
Tebuconazole	Brown planthopper	*Nilaparvata lugens*	*Acinetobacter* sp.	Song et al., 2021
DDT	Diamondback moth	*Plutella xylostella*	*Bacillus thuringiensis* and *Saccharopolyspora spinosa*	Sarfraz and Keddie, 2005
Imidacloprid	Honeybee Fruit fly Fruit fly Whitefly Bed bug	*Apis mellifera* *Bactrocera tau* *Drosophila melanogaster* *Bemisia tabaci* *Cimex hemipterus*	*Bifidobacterium* sp., *Lactobacillus* sp., *Klebstella oxytoca, Pantoea agglomerans, Staphylococcus* sp. *Lactobacillus* sp., *Rickettsia* sp., *Frischella* sp. *Wolbachia* sp., *Yersinia* sp., *Bacillus* sp., and *Acetobacter* sp.	Kontsedalov et al., 2008; Prabhakar et al., 2008; Chmiel et al., 2019; Rouzé et al., 2019; Alberoni et al., 2021; Soh and Veera Singham, 2022
Atrazine	Jewel wasp	*Nasonia vitripennis*	*Serratia marcescens* and *Pseudomonas protegens*	Wang G. -H. et al., 2020
Chlorpyriphos	Diamondback moth	*Plutella xylostella*	*Enterobacteriales* sp., *Vibrionales* sp., *Pseudomonadales* sp., *Xanthomonadales* sp., and *Lactobacillales* sp.	Xia et al., 2013
Fipronil	Diamondback moth Honeybee	*Plutella xylostella* *Apis mellifera*	*Enterobacteriales* sp., *Vibrionales* sp., *Pseudomonadales* sp., *Xanthomonadales* sp., *Lactobacillales* sp., *Bifidobacterium* sp., *Alphaproteobacteria* sp., *Gammaproteobacteria* sp., and *Lactobacillus* sp.	Xia et al., 2013; Rouzé et al., 2019; Paris et al., 2020
Pyraclostrobin	Honeybee	*Apis mellifera*	*Gilliamella* sp. and *Lactobacillus* sp.	DeGrandi-Hoffman et al., 2017
Abamectin	Parasitic wasps	*Eretmocerus mundus, Eretmocerus eremicus*, and *Encarsia formosa*	*Arthrobacter* sp.	Fernández et al., 2019
Thiamethoxam	Whitefly Honeybee	*Bemisia tabaci* *Apis mellifera*	*Delftia* sp., *Rickettsia* sp., *Bifidobacterium* sp., *Lactobacillus* sp. *Alphaproteobacteria* sp., and *Gammaproteobacteria* sp.	Kontsedalov et al., 2008; Xie et al., 2012; Rouzé et al., 2019; Paris et al., 2020
Deltamethrin	Diamondback moth Mosquitos Cotton aphid	*Plutella xylostella* *Anopheles albimanus* *Aphis gossypii*	*Enterococcus mundtii, Carnobacterium maltaromaticum, Bacillus* sp., *Buchner* sp., *Pseudomonas* sp., *Pantoea agglomerans* and *Pseudomonas fragi*	Li et al., 2017; Dada et al., 2019; Shang et al., 2021b
Coumaphos	Honeybee	*Apis mellifera*	*Bifidobacterium* sp. and *Lactobacillus* sp.	Rouzé et al., 2019
Malathion	Fruit fly	*Bactrocera tau*	*Klebstella oxytoca, Pantoea agglomerans*, and *Staphylococcus* sp.	Prabhakar et al., 2008
Cypermethrin	Tobacco cutworm or cotton leafworm	*Spodoptera litura*	*Clostridium botulinum, Clostridium butyricum*, and *Pseudomonas putida*	Karthi et al., 2020
Phoxim	Silkworm	*Bombyx mori*	*Enterobacter cloacae, Staphylococcus* sp., *Methylobacterium* sp., and *Aurantimonadaceae* sp.	Li F. et al., 2020
Beta-cypermethrin	Cockroach	*Blattella germanica*	*Lactobacillus* sp., *Metarhizium anisopliae, Parabacteroides* sp., *Lachnoclostridium* sp., and *Tyzzerella* sp.	Zhang and Yang, 2019; Zhang J. et al., 2022
Carboxamide	Honeybee	*Apis mellifera*	*Alphaproteobacteria* sp. and *Gammaproteobacteria* sp.	Paris et al., 2020
Phosphine	Red flour beetle	*Tribolium castaneum*	*Bacillus subtilis, Staphylococcus* sp., *saprophyticus* sp., *Enterobacter* sp., *Lysinibacillus fusiformis, Klebsiella pneumonia*, and *Achromobacter* sp.	Gowda et al., 2021
Trichlorphon	Oriental fruit fly	*Bactrocera dorsalis*	*Citrobacter freundii*	Guo et al., 2017
Endosulfan	Fruit fly	*Bactrocera tau*	*Klebstella oxytoca, Pantoea agglomerans*, and *Staphylococcus* sp.	Prabhakar et al., 2008
Temephos	Asian malaria mosquito	*Anopheles stephensi*	*Pseudomonas* sp., *Aeromonas* sp., *Exiguobacterium* sp., and *Microbacterium* sp.	Soltani et al., 2017
Permethrin	Mosquitos African malaria mosquito	*Anopheles albimanus* *Anopheles gambiae*	*Pantoea agglomerans* and *Pseudomonas fragi.* *Sphingobacterium, Lysinibacillus* and *Streptococcus*	Dada et al., 2019 Omoke et al., 2021
Alphacypermethrin	Mosquitos	*Anopheles albimanus*	*Pantoea agglomerans* and *Pseudomonas fragi*	Dada et al., 2019
Thiacloprid	Honeybee	*Apis mellifera*	*Enterococcus faecalis, Snodgrassella alvi, Bartonella apis, Frischella perrara, Lactobacillus kunkeei, Frischella* sp., *Bifidobacterium asteroids*, and *Gilliamella apicola*	Dickel et al., 2018; Alberoni et al., 2021; Cuesta-Maté et al., 2021
Fenitrothion	Bean bug	*Riptortus pedestris*	*Burkholderia* sp.	Itoh et al., 2018
Pyriproxyfen	Silkworm Whitefly	*Bombyx mori* *Bemisia tabaci*	*Burkholderia* sp., *Rhizobia* sp., *Rickettsia* sp., *Caulobacter* sp., *Sphingobacteria* sp., and *Enterobacteria* sp.	Kontsedalov et al., 2008; Lu et al., 2022
Chlorpyriphos	Diamondback moth	*Plutella xylostella*	*Enterococcus* sp., *Enterobacter* sp., and *Serratia* sp.	Gurr et al., 2018
Acetamiprid	Honeybee Whitefly	*Apis mellifera* *Bemisia tabaci*	*Snodgrassella alvi, Bartonella apis, Frischella perrara, Lactobacillus kunkeei, Bifidobacterium asteroids, Gilliamella apicola*, and *Rickettsia* sp.	Kontsedalov et al., 2008; Cuesta-Maté et al., 2021
Spinosyns	Diamondback moth	*Plutella xylostella*	*Bacillus thuringiensis* and *Saccharopolyspora spinosa*	Sarfraz and Keddie, 2005
Pendimethalin	Ground beetle	*Pterostichus melas*	*Enterobacter* sp., *Pseudomonas* sp., *Pantoea* sp., and *Paracoccus* sp.	Giglio et al., 2021
Sulfoxaflor	Cotton aphid	*Aphis gossypii*	*Buchner* sp. and *Arsenophonus* sp.	Shang et al., 2021b
Avermectin	Gypsy moth	*Lymantria dispar asiatica*	*Weissella* sp., *Lactobacillus* sp., *Pseudomonas* sp., *Candida* sp., *Tausonia* sp., *Chaetomium* sp., *Diutina* sp., and *Alternaria* sp.	Zeng et al., 2020
Buprofezin	Small brown planthopper	*Laodelphax striatellus*	*Wolbachia* sp. and *Rickettsia* sp.	Li et al., 2018
Boscalid	Honeybee	*Apis mellifera*	*Gilliamella* sp. and *Lactobacillus* sp.	DeGrandi-Hoffman et al., 2017
Carbaryl	Fall armyworm	*Spodoptera frugiperda*	*Bacillus thuringiensis* and *Varimorpha necatrix*	Fuxa and Richter, 1990
Methyl parathion	Fall armyworm	*Spodoptera frugiperda*	*Bacillus thuringiensis* and *Varimorpha necatrix*	Fuxa and Richter, 1990
Spiromesifen	Whitefly	*Bemisia tabaci*	*Rickettsia* sp.	Kontsedalov et al., 2008
Glyphosate	Colorado potato beetle	*Leptinotarsa decemlineata*	*Agrobacterium* sp., *Ochrobactrum* sp., *Rhodobacter* sp., *Rhizobium* sp., and *Acidovorax* sp.	Gómez-Gallego et al., 2020
Guadipyr	Silkworm	*Bombyx mori*	*Pseudomonas* sp. and *Curvibacter* sp.	Hou et al., 2021
Lufenuron	Formosan subterranean termite	*Coptotermes formosanus*	*Pseudomonas aeruginosa, Serratia marcescens*, and *Bacillus thuringiensis*	Wang et al., 2013
Fenitrothion	Bed bug	*Cimex hemipterus*	*Wolbachia* sp., *Yersinia* sp., and *Bacillus* sp.	Soh and Veera Singham, 2022
Spiromesifen	Whitefly	*Bemisia tabaci*	*Rickettsia* sp.	Kontsedalov et al., 2008

**FIGURE 2:**
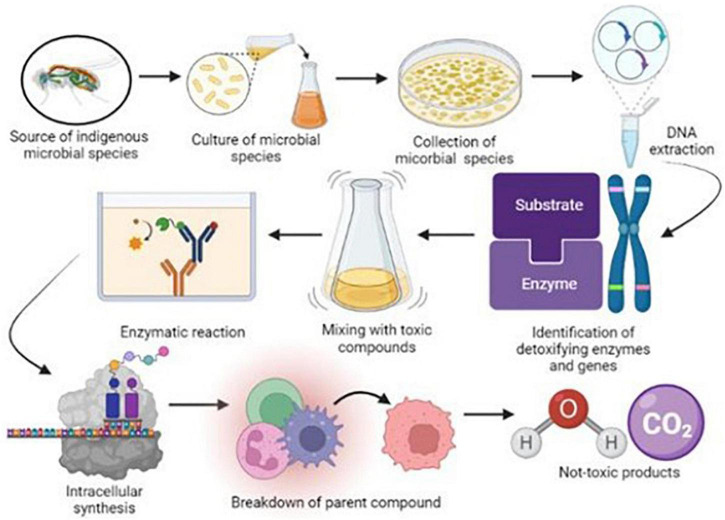
Schematic diagram of isolation of insect gut microbial species and their functions in biodegradation of environmental pollutants.

Insect digestive systems have a robust defensive system mainly equipped with various microbial species such as bacteria, fungi, archaea, and protozoa (Chen et al., 2021). In a recent study, the isolation of various microbial species in the digestive tract of worker honeybees (*Apis mellifera*). Results of this study demonstrated that nine species of bacteria from various genera were isolated; five belonged to *Snodgrassella alvi*, *Gilliamella apicola*, two species were from *Lactobacillus*, and one from *Bifidobacterium* (Douglas, 2018). Various microbial communities allow insects to tolerate or reject toxic compounds through various metabolic processes and develop a peritrophic medium composed of chitin microfibrils and a protein-carbohydrate medium (Kamalakkannan et al., 2017; Rumbos et al., 2018). This peritrophic medium plays a pivotal role in the development of resistance against chemicals due to some prominent features such as releasing digestive enzymes, availability of nutrients and providing protection to epithelial cells from external microbes and toxins through a semipermeable membrane (Puri et al., 2022; Siddiqui et al., 2022). These physiological obstacles between the lumen and epithelium actively contribute to the defense mechanism and minimize the activation of pesticides on the host rather than reducing microbial load in the gut (Chen et al., 2022; Figure 2).

Recently, a laboratory experiment was conducted to investigate organophosphate pesticide resistance in a serious rice pest, *Cletus punctiger* by the gut symbiont. Results of this study demonstrated that a rice bug effectively degraded organophosphate fenitrothion by *Burkholderia* bacterial specie *via* oral infection and stayed it in the midgut part of the rice bug. The degradation of fenitrothion by the isolating bacterial species from the midgut revealed that gut microbiomes are highly capable of degrading pesticides in insects, and insect gut symbiosis plays a significant role in the development of resistance against fenitrothion in the host rice bug (Ishigami et al., 2021). In another study, the resistance of stored grain products against phosphine fumigation was studied. Four major stored grain pests (*Rhyzopertha dominica*, *Sitophilus granaries, Tribolium castaneum*, and *Trogoderma granarium*) were reared under laboratory conditions for up to seven generations. Results of this study indicated that insect gut symbiosis develops resistance in all pests. Regarding their level of resistance, *Rhyzopertha dominica* was highly resistant followed by *Tribolium castaneum, Trogoderma granarium*, and *Sitophilus granaries.* Although this study concluded that phosphine tablets are excessively applied to manage stored products and are considered very efficient against various stored grain pests, the development of resistance may lead to a serious failure of their applications (Wakil et al., 2021).

An investigation was carried out to study the role of gut microbiota in developing resistance against various insecticides in the laboratory and open field conditions in the larvae of *Spodoptera frugiperda*. The insect pests were collected from various corn fields in five Brazilian states. In a metagenomic experiment and 16S rRNA analysis, the isolation of bacterial species from insect gut in the selective medium was achieved. The maximum growth of microbial species in insecticides was observed, and it was found that all microbes utilized it as a sole source of carbon and energy. This study indicated that bacteria isolated from field larvae grew better and degraded insecticides more efficiently than those collected from laboratory-selected strains. However, this study concluded that due to the high efficiency and diversity of insect gut microbes in the field, larval insects are more capable of degrading pesticides and showed high resistance (Gomes et al., 2020).

A study was conducted to evaluate the resistance of chlorpyriphos in diamondback moths (*Plutella xylostella*) by insect gut microbiota. In this investigation, three bacterial species from insect guts such as *Enterococcus* sp., *Enterobacter* sp., and *Serratia* sp. were isolated and examined for their role in detoxifying chlorpyriphos and developing resistance in diamondback moths. Results of this study indicated that *Enterococcus* sp. increased resistance against the most widely used insecticide, chlorpyriphos. At the same time, *Serratia* sp. reduced resistance in the diamondback moth and for *Enterobacter* sp. no effect was observed. In addition, this study explained that *Enterococcus* sp., vitamin C and acetylsalicylic acid increased the regulation of antimicrobial peptides, which played a crucial role in the development of insecticide resistance (Xia et al., 2018). In another study, Wang et al. (2021) explained that insect gut microbial species play a significant role in insecticide deltamethrin resistance in *Aedes albopictus.* Additionally, experimental results indicated that by full-length 16S rRNA analysis, two bacterial species were collected from insect guts such as *Serratia oryzae* and *Acinetobacter junii* and investigated their growth in six kinds of growth media in biotic and abiotic conditions. Further, they observed that both symbiotic bacteria are mainly facultative in an anaerobic environment. Moreover, this study explained that insect symbiotic bacterial species actively promoted insect resistance against insect pesticides.

### Molecular mechanism of resistance against pesticides by insect gut microbiota

To identify the complete profile and total biodiversity of microbial communities in insect gut microbiota and polluted sites, modern molecular biological approaches including clone libraries, probes, reverse sample genome probing, fluorescence *in situ* hybridization, community profiling or DNA fingerprinting, next-generation sequencing and pyrosequencing provide a more significant explanation as compared to the conventional biological tools (Ahmad et al., 2022b). Various functional parts of an insect’s gut microbes, such as enzymes and genes, are responsible for developing pesticide resistance in insects (Bhatt et al., 2022b). Metagenomic analysis was performed to identify major microbial species in the gut of a honeybee (*Apis mellifera*) and their functional roles in developing resistance. This study’s results revealed that insect gut microbe gene contents (*Gilliamella apicola*) are related to various host-dependent symbiotic functions. Moreover, as evidenced by the case of pectin breakdown by *G. apicola*, genetic variations are related to functional variations. The glycoside hydrolase and polysaccharide lyase enzyme families discovered in the honeybee metagenome are depicted with their respective cleavage sites on the schematic of the pectin molecule (Engel and Moran, 2013). In another study, an investigation of gut microbial species from three diamondback moth larvae was carried out to study prothiofos resistance. Findings from 16S rRNA showed that the bacterial community from the prothiofos-resistant larval gut was more diversified. In addition, the secretion of chitinase enzymes from the population of insect gut bacteria significantly contributed to host antagonism against entomopathogens and nutrition (Indiragandhi et al., 2007). Pesticide-resistance cockroach species such as German cockroaches, American cockroaches, and Oriental cockroaches are rich sources of insect gut microbiota and play a crucial role in insect physiology (Zhang X. C. et al., 2022). For example, the effect of beta-cypermethrin resistance development in cockroaches (*Blattella germanica*) by the gut microbial population and their genetic association with host growth was investigated. Results of 16S rRNA gene sequencing and metagenomics indicated that *Lactobacillus* spp. were abundantly present in the foregut and midgut of cockroaches. In addition, carbohydrate-active enzymes actively contribute to developing resistance, insect growth, and fitness (Zhang and Yang, 2019). However, modern molecular biological tools efficiently describe the microbial interaction with the host and external pathogens. Moreover, in the future, these approaches could be applied to using and managing environmental bioprocesses through knowledge-based control.

### Role of gut microbiota for biodegradation of pesticides

**FIGURE 3:**
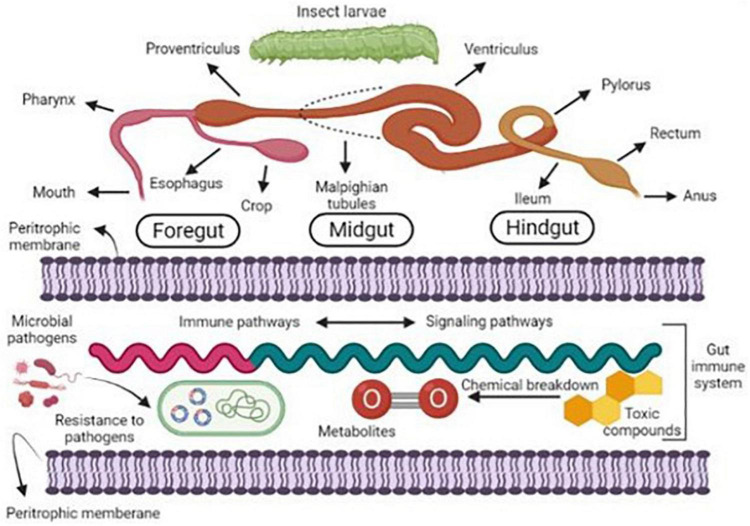
Graphical representation of gut microbes’ development of resistance against pesticides.

In many insect species, resistance to pesticides has been confirmed. It has been found that they are very beneficial for degrading toxic compounds due to their digesting abilities (Schmidt and Engel, 2021). The degradation of pesticides depends on various factors such as microbial remediation and the chemical hydrolysis process, which are additionally correlated with many physiological properties such as pH, temperature, organic matter, and moisture content (Bhatt et al., 2022b). However, the insect gut provides a favorable environment for developing diverse microbial communities. Hence, they efficiently deliver many promising facilities to their host (Shan et al., 2021). Symbiotic microbial species isolated from insect gut can perform in extreme environmental conditions to degrade pesticides and other emerging pollutants (Francis and Aneesh, 2022; Figure 3).

Recently, Wang et al. (2022) investigated the degradation of various pesticides by isolating microbial species from stored grain pests and studied the resistance mechanism. In this experiment, from multiple locations, adults of different stored grain pests (*Sitophilus oryzae*, *Cryptolestes ferrugineus*, and *Rhyzopertha dominica*) were collected and isolated as five bacterial species. Results of this study indicated that all screened bacterial species could degrade deltamethrin, malathion, and pirimiphos-methyl efficiently and use their residues as a source of carbon and energy, which are favorable for their growth. Additionally, this study revealed that when bacterial species are treated with 0.5–10 mg/kg of malathion, pirimiphos-methyl, and 0.3–0.75 mg/kg of deltamethrin, gnotobiotic reinoculation and their survival rates in the host are significantly increased, which implies that development of insecticide resistance highly depends on concentration rate. Moreover, this study also explained that the *in vitro* biodegradation of pesticides through gut bacteria was not entirely consistent with their *in vivo* operation in host pesticide resistance, which suggested that instead of direct degradation of pesticides, other physiological and morphological processes are also responsible for pesticide tolerance or resistance.

In another study, insects of Orthoptera and Dermaptera were collected from various sites. Fourteen bacterial species were isolated for the biodegradation of deltamethrin from a polluted environment. All the bacterial species were analyzed by 16SRNA and identified as *Poecilimon tauricola*, *Locusta migratoria*, *Gryllus bimaculatus*, *Forficula Auricularia, Pseudomonas aeruginosa*, *Stenotrophomonas maltophilia*, *Bacillus atrophaeus*, *Acinetobacter lwoffii*, *Rhodococcus coprophilus*, *Brevundimonas vesicularis*, *Pseudomonas syringae*, *Yersinia frederiksenii*, *Bacillus licheniformis*, *Enterobacter intermedius*, and *Serratia marcescen.* In addition, this study explained that eleven of them were gram-negative bacteria, and three were gram-positive bacteria, potentially deleting deltamethrin up to 100 mg/L. This study concluded that insecticide-tolerated gut microbiota is enriched with nutrients and is considered a powerful tool for the remediation of various kinds of pollutants from the ecosystem (Özdal and Algur, 2020). An indigenous rod-shaped gram-negative bacterium was isolated from the digestive tract of a grasshopper (*Poecilimon tauricola*) for the potential biodegradation of α-endosulfan and α-cypermethrin. By morphological, physiological, and 16S rRNA sequence analysis, the bacterial strain was characterized as *Acinetobacter schindler* and named B7. This study demonstrated that when the bacterial strain was treated with 100 mg/L of both pesticides in a glucose-mediated non-sulfur medium, significant growth of the bacterial strain was observed. Additionally, this study showed that within 10 days, the bacterial strain was capable of degrading 67.31 and 68.4% of α-endosulfan and α-cypermethrin, respectively (Ozdal and Algur, 2022).

Organophosphates are an influential group of pesticides that are excessively applied to control insect pest infestations across several agricultural and horticultural crops. The residues of this group are very toxic to the environment and spread many diseases to non-target organisms. Therefore, it is essential to remove their residues from the ecosystem by a potential degradation method. Recently, a study was conducted on the effective biodegradation of organophosphate pesticides, chlorpyriphos and polyethylene, by isolating insect gut microbial species. This study isolated four potential bacterial species: *Bacillus licheniformis*, *Pseudomonas cereus*, *Pseudomonas putida*, and *Bacillus subtilis*, from the gut of the citrus mealybug (*Planococcus citri).* Results of this study revealed that all symbiotic bacterial species utilized chlorpyriphos and polyethylene as a sole source of carbon and energy and enhanced their growth and enzymatic activity. Findings of the degradation experiment showed that after the treatment of 45 days, a satisfactory reduction of polyethylene weight was noticed, and scanning electron microscope analysis suggested that a biofilm formation around the polyethylene sheet by bacterial isolates was also observed. While in the case of chlorpyriphos, results indicated that after 21 days, significant degradation was observed in soil and water. In addition, this study revealed that *Pseudomonas cereus* and *Pseudomonas putida* have more potential to degrade both pollutants in diverse environmental conditions (Ibrahim et al., 2021).

To degrade clothianidin residues in an open environment, seven bacterial species such as *Edwardsiella* sp., two *Serratia* sp., *Rahnella* sp., *Pantoea* sp., *Hafnia* sp., and *Enterobacter* sp. were isolated from the digestive tract of the honeybee. To examine the growth of all bacterial strains, they were treated with various concentrations of clothianidin as a sole source of carbon and energy. They found that all bacterial strains provide satisfactory growth up to 10 ppb of clothianidin. Results of the degradation experiment showed that within 3 days, all endogenous bacterial strains noticed complete degradation of clothianidin (El Khoury et al., 2022).

For the biodegradation of multi-pesticides such as chlorpyriphos, cypermethrin, malathion, quinalphos, and triazophos, 13 indigenous microbial species were isolated from the gut of the cotton bollworm (*Helicoverpa armigera*) and tested for their degradation efficiency. After physiochemical, morphological and 16S rRNA sequence analysis, all bacterial strains were identified as *Bacillus pumillis* CL1, *Enterococcus casseliflavus* CL2, *Bacillus subtilis* CL3, *Rhodococcus* sp. CL4, *Pseudomonas* sp. CL5, *Staphylococcus* sp. CL6, *Pseudomonas aeruginosa* CL7, *Proteus vulgaris* HL1, *Cellulosimicrobium cellulans* HL2, *Klebsiella oxytoca* HL3, *Bacillus subtilis* HL4, *Stenotrophomonas maltophilla* HL5, and *Pseudomonas* sp. HL6. Results of this study indicated that strains CL2 and CL4 provided more rapid growth in the presence of malathion and chlorpyriphos in a mineral salt medium. Gas chromatography and mass spectrometry analysis revealed that strain CL4 has the potential to degrade 44% of chlorpyriphos and strain CL2 was capable to degrade 26% of chlorpyriphos and 57.1% of malathion in mineral salt medium (Madhusudhan et al., 2021). In another study, a symbiotic gut bacterium, *Citrobacter* sp. (CF-BD), was isolated from the digestive tract of tephritid fruit flies (*Bactrocera dorsalis*) and investigated their degradation efficiency against trichlorphon insecticide. Results of this study showed that insect gut microbiota plays a vital role in developing resistance against toxin chemicals and efficiently degrade trichlorphon into metabolites. This study also explains that various hydrolase genes were identified in the bacterial isolate CF-BD. In the presence of trichlorphon, the maximum gene expression was observed, and it was found that these critical genes play a crucial role in developing resistance in tephritid fruit flies (Cheng et al., 2017).

### Screening of gut microbial enzymes for biodegradation of pesticides

In the insect body system, various potential natural enzymes are linked with different biological processes and play a vital role in toxic detoxifying substances in target sites (Lin et al., 2015; Naik et al., 2018; Bhandari et al., 2021). Based on some previous reports, it is found that resistance or tolerance of pesticides is interlinked with these biochemical processes which are held in the insect body and sensitivity of pesticides which are further degraded by metabolic enzymes such as hydrolase, esterase, laccase, acetylcholinesterase, carboxylesterase, glutathione S-transferase, cytochrome P450 and many more (Ismail, 2020; Clark et al., 2021; Yang Y. -X. et al., 2021; Ahmad et al., 2022b; Siddiqui et al., 2022). Insect pests’ resistance against different pesticides by insect gut microbial enzymes was reported (Table 2). In the midgut of the tobacco budworm (*Heliothis virescens*), many essential enzymes were purified, such as 58 proteinases, four cadherins, 13 aminopeptidases, and five alkaline phosphatases. Other putative detoxification enzymes include 20 cytochrome P450 oxidases, 11 glutathione S-transferases, nine esterase’s, and 15 cytochrome oxidases. These enzymes contributed to insect physiology and reduced the toxicity of pesticides (Zhu et al., 2011).

**TABLE 2:** Functions of insect enzymes in detoxification of pesticides and insect physiology.

Insect common name	Insect scientific name	Name of pesticide	Name of enzyme	Functions	Reference
Spongy moth	*Lymantria dispar*	Methidathion	Superoxide dismutase, catalase, glutathione peroxidase	Develop defense mechanism and protect from oxidative stress	Aslanturk et al., 2011
Fall armyworm	*Spodoptera frugiperda*	Organophosphate insecticides	Alkaline phosphatase, esterase, glutathione S-transferase, aminopeptidase, and proteinase	Resistance, detoxification of pesticides	Zhu et al., 2015
Parasitic wasps	*Eretmocerus mundus, Eretmocerus eremicus* and *Encarsia formosa*	Abamectin	Esterases	Resistance, support to gut microbes, play key role in insect biology, ecology and behavior	Fernández et al., 2019
Colombian mosquito	*Aedes aegypti*	Pyrethroid insecticides	Esterases and oxidases	Resistance and mutation development	Granada et al., 2021
Whitefly	*Bemisia tabaci*	Neonicotinoid insecticides	Cytochrome P450	Insecticide resistance, support to symbiotic bacteria	Barman et al., 2021
Honeybee	*Apis mellifera*	Flumethrin	Catalase	Resistance, increased immunity to pathogens and improvement of detoxification genes (*GST, Hymenoptaecin, Defensin1, Catalase, GAPDH*)	Yu et al., 2021
Greater Wax Moth	*Galleria mellonella*	Malathion	Esterase and glutathione S-transferase	Resistance, detoxification of malathion and development of complex biological products	Serebrov et al., 2006
Yellow fever mosquito	*Aedes aegypti*	Permethrin	Cytochrome P450 monooxygenases	Insecticide resistance, perform multiple biological functions and metabolize pesticide	Somwang et al., 2011; David et al., 2014
Yellow fever mosquito	*Aedes aegypti*	Deltamethrin	Cytochrome P450	Metabolic resistance	Faucon et al., 2015
Diamondback moth	*Plutella xylostella*	Fenvalerate, fipronil, flufenoxuron and monocrotophos	Hydrolases, transferases and oxygenase’s	Detoxification of pesticides and resistance development	Mohan and Gujar, 2003
Yellow fever mosquito	*Aedes aegypti*	Glyphosate and alpha pyrene	Cytochrome P450 monooxygenases, glutathione S-transferases and carboxy/cholinesterase	Resistance, improvement of detoxification genes and development of biological products	Riaz et al., 2009
Brown planthopper	*Nilaparvata lugens*	Acephate, thiamethoxam and buprofezin	Esterases, glutathione S-transferases and mixed-function oxidases	Resistance	Malathi et al., 2017
Housefly	*Musca domestica*	Diazinon	Cytochrome P450	Resistance and role in insect biology, ecology, and behavior	Cariño et al., 1994
African malaria mosquito	*Anopheles gambiae*	Bendiocarb	Cytochrome P450	Resistance and detoxification of pesticide	Edi et al., 2014
Boisduval	*Tetranychus cinnabarinus*	Abamectin and fenpropathrin	Carboxylesterases, mixed function oxidase, glutathione S-transferases, and hydrolases,	Resistance	Lin et al., 2009
Green peach aphid	*Myzus persicae*	Neonicotinoid insecticides	Cytochrome P450	Resistance and improve detoxification genes	Puinean et al., 2010
Yellow fever mosquito	*Aedes aegypti*	Organophosphate, carbamate and some pyrethroid insecticides	α and β Esterases, mixed-function oxidases, glutathione-S-transferase, acetylcholinesterase, and insensitive acetylcholinesterase	Resistance, support to gut microbes, play key role in insect biology, ecology, and behavior	Flores et al., 2006
Yellow fever mosquito	*Aedes aegypti*	DDT and deltamethrin	Glutathione S-transferase and dehydrochlorinase	Resistance and detoxification of pesticides	Lumjuan et al., 2011
Annual bluegrass weevil	*Listronotus maculicollis*	Bifenthrin	Cytochrome P450 monooxygenases, glutathione S-transferases, and carboxylesterases	Detoxification, resistance and development of biological products	Ramoutar et al., 2009
Yellow fever mosquito	*Aedes aegypti*	Permethrin, temephos and atrazine	Cytochrome P450 monooxygenases	Resistance	Poupardin et al., 2008
Australian sheep blowfly	*Lucilia cuprina*	Organophosphate insecticides	Carboxylesterases and acetylcholinesterase	Resistance, detoxification of insecticides and provide protection from external pathogens	Jackson et al., 2013
Green peach aphid	*Myzus persicae*	Imidacloprid, acetamiprid and cyhalothrin	Acetylcholinesterase, carboxylesterase, glutathione-S-transferase, and mixed-function oxidase, superoxide dismutase, catalase, peroxidase, amylase	Food digestion, resistance development, breakdown of pesticide compounds and provide protection from external pathogens	Cai et al., 2021
Cotton bollworm	*Helicoverpa armigera*	Esfenvalerate, indoxacarb, emamectin benzoate and chlorantraniliprole	P450 enzymes	Resistance and detoxification of pesticides	Wang et al., 2018
Australian cotton bollworm	*Helicoverpa armigera*	Fenvalerate	Cytochrome P450 monooxygenase and carboxylesterases	Resistance and provide protection from external pathogens	Joußen et al., 2012
Red spider mite	*Tetranychus urticae*	Abamectin	Cytochrome P450	Resistance	Riga et al., 2014
Asian malaria mosquito	*Anopheles stephensi*	Pyrethroid and organophosphate insecticides	Cytochrome P450s, esterase’s, glutathione S-transferases and acetylcholine esterase	Resistance and detoxification of pesticides	Safi et al., 2017
Migratory locust	*Locusta migratoria*	Carbaryl, malathion, and deltamethrin	Cytochrome P450 monooxygenases	Resistance	Guo et al., 2012
Oriental fruit fly	*Bactrocera dorsalis*	Fenitrothion	Acetylcholinesterase	Resistance and support to detoxification genes	Hsu et al., 2006
African malaria mosquito	*Anopheles gambiae*	Deltamethrin	Cytochrome P450 enzymes	Resistance	Yahouédo et al., 2017
White-backed planthopper	*Sogatella furcifera*	Imidacloprid, deltamethrin and triazhophos	Cytochrome P450 enzymes	Detoxification of pesticides and development of resistance	Zhou et al., 2018
Bed bug	*Cimex lectularius*	Deltamethrin	Cytochrome monooxygenase, esterase’s, glutathione S-transferase, and carboxylesterase	Resistance	Gonzalez-Morales and Romero, 2019
Cowpea aphid	*Aphis craccivora*	Thiamethoxam	Glutathione S-transferase and mixed function oxidases	Resistance	Abdallah et al., 2016
Small brown planthopper	*Laodelphax striatellus*	Chlorpyriphos and dichlorvos	Alkaline phosphatase, carboxylesterase, acetylcholinesterase, acid phosphatase, glutathione S-transferase and cytochrome P450 monooxygenase	Resistance and detoxification of pesticides	Wang et al., 2010

An investigation was conducted to understand the enzymatic molecular mechanism for biodegradation of chlorpyriphos, glyphosate, phoxim, and esfenvalerate. In this study, 263 bacterial colonies were isolated from the gut of a cricket (*Teleogryllus occipitalis*), cultured individually, and examined for their degradation efficiency. Based on morphological, physiological, and 16S rRNA analysis and found that 55 bacteria species showed a high resemblance to 28 genera. Among these 55 bacterial species, 18 have the potential to degrade 50%, and six were able to degrade 70% of chlorpyriphos at an initial concentration of 400 mg/L within 1 day of incubation in a mineral salt medium. In addition, purification of extracellular hydrolase enzymes was studied in these isolates and found that free cells and hydrolase enzymes play a crucial role in the degradation of chlorpyriphos, glyphosate, phoxim, and esfenvalerate. A carboxyl esterase enzyme was purified from the mosquito gut bacteria (*Escherichia coli*) and studied for its effectiveness in degrading malathion. The results of the degradation experiment revealed that these carboxylesterase enzymes could efficiently degrade more than 80% of malathion. This study concluded that due to their rapid degradation ability, superior stability, and high activity, these enzymes could further degrade other organophosphate pesticides that contaminate the environment (Zhang et al., 2004).

In another study, a study was carried out on the biodegradation of organophosphate and pyrethroid pesticides from the contaminated environment to evaluate the resistance mechanism in insect pests (*Helicoverpa armigera*). In this study, a yeast (*Pichia pastoris* HaGST-8) was isolated from the insect gut and purified glutathione-S-transferase enzymes to detoxify chlorpyriphos dichlorvos and cypermethrin up to a concentration of 2–15 mg/L. Results of this study revealed that these enzymes have the potential to degrade all organophosphate pesticides completely and cypermethrin partially (53%) in an aqueous solution. Moreover, this study suggested that isolated yeast provides satisfactory growth in all pesticides at higher concentrations (200–400 mg/L) and concluded that these purified enzymes could be further utilized to degrade other pesticide groups such as organophosphates, carbamates, pyrethroids, organochlorines, and organophosphates from food, soil, and water resources (Labade et al., 2018). Two potential enzymes, such as cytochrome P450 monooxygenase and esterase, were purified from an insect gut (*Aedes aegypti*) bacterium and characterized for their efficiency in degrading propoxur and naled insecticides. Results of this study indicated that both types of enzymes play a crucial role in the degradation of pesticides and are further metabolized into non-toxic substances. This study concluded that insect gut symbiotic bacteria and their associated enzymes reduced the toxicity of pesticides, enhanced resistance, and played an essential role in the digestion of food (Scates et al., 2019).

The potential and purification of three enzymes such as hydrolases, transferases, and oxygenase’s, were purified from the insect (*Plutella xylostella*) gut bacterium (*Bacillus thuringiensis var. kurstaki* HD-1) and investigated their role in the development of resistance and degradation of insecticides such as fenvalerate, fipronil, and flufenoxuron. This study demonstrated that all detoxifying enzymes were responsible for developing resistance against the diamondback moth insecticides and metabolizing them into less toxic substances (Mohan and Gujar, 2003). However, it has been proven that a diverse number of prokaryotic and eukaryotic microbial species in the insect gut and their associated enzymes actively contribute to the degradation of various pesticides and metabolize them into less toxic substances. These less toxic substances were further utilized by microbiota as a sole source of carbon, sulfur, and energy and play a key role in insect physiology (Mohammadi et al., 2021).

## Microbial metabolic pathways

The high application of pesticides to control various pests has produced long-term hazardous residual pollution in the ecosystem (Shahid et al., 2021). A lot of insect pest species depend on insect gut microbial species to attain nutrients, in defense mechanisms, exploit novel food resources and undergo metabolization of parent compounds into their intermediates (Chen et al., 2020). Various microbial species break down parent compounds into their metabolites, which pose the same or higher toxicity than the parent compound and disturb the environmental equilibrium.

**FIGURE 4:**
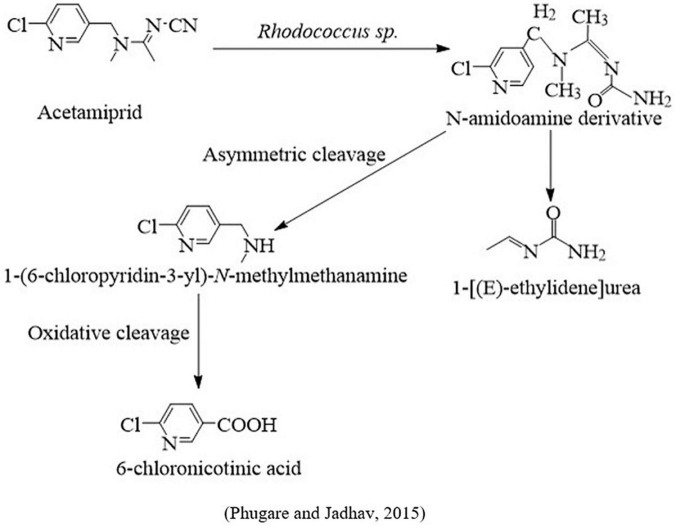
Microbial metabolic pathway of acetamiprid degradation by insect gut microbiota.

**FIGURE 5:**
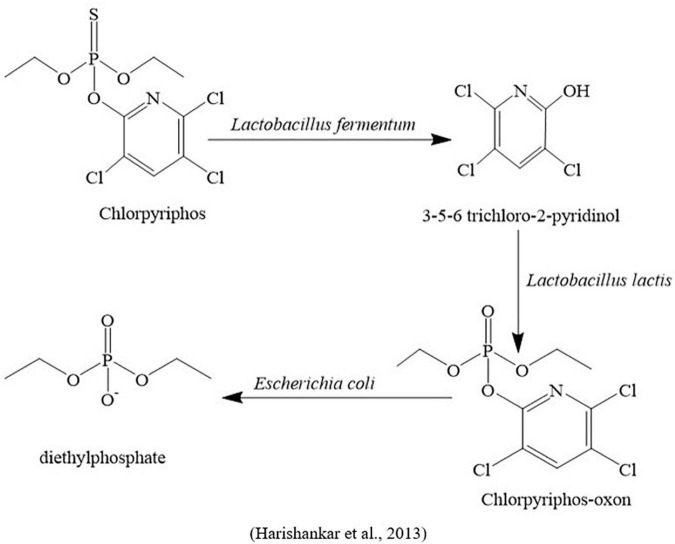
Microbial metabolic pathway of chlorpyriphos degradation by insect gut microbiota.

An acetamiprid-degrading bacterium that provides the highest growth on a mineral salt medium was isolated and, based on morphological, physiological, 16S rRNA, and BLAST analysis, identified as *Rhodococcus* sp. BCH2. This study showed that the acetamiprid was rapidly metabolized into its three metabolites, such as *N*-amidoamide derivative, 1-(6-chloropyridin-3yl)-*N*-methylmethanamine, and 6-chloronicotinic acid. Additionally, toxicological effects of the parent compound and their metabolites on silkworm (*Bombax mori*) concerning genotoxicity, antioxidant enzymes, lipid peroxidation, and protein oxidation were also investigated. This study suggests that the parental molecule has more hazardous effects on insect physiology than its derivatives (Phugare and Jadhav, 2015; Figure 4). In another study, five intestinal bacterial species were screened to understand the chlorpyriphos biodegradation mechanism. After characterization of physiological and morphological properties, bacterial species were identified as *Lactobacillus lactis*, *Lactobacillus fermentum*, *Lactobacillus plantarum*, *Escherichia coli*, and *Enterococcus faecalis.* Plate assay findings indicated that three of them (*Lactobacillus fermentum, Escherichia coli*, and *Lactobacillus lactis*) could provide maximum growth even using higher dosages (>1400 μg/L), while the other two bacterial species (*Lactobacillus plantarum* and *Enterococcus faecalis*) were able to grow using less concentrations (100 and 400 μg/L), respectively. Based on growth parameters, the best three bacterial species were investigated to degrade chlorpyriphos and found that *L. fermentum* was able to degrade 70% of chlorpyriphos and generate one metabolite named 3,5,6-trichloro-2-pyridinol, *L. lactis* degraded 61% of chlorpyriphos into chlorpyrifos oxon, and *E. coli* provided less chlorpyriphos degradation (16%) and breakdown in chlorpyrifos oxon and diethyl phosphate (Figure 5).

**FIGURE 6:**
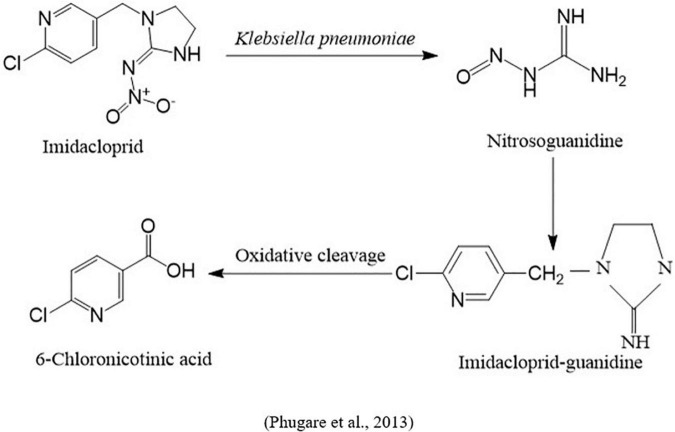
Microbial metabolic pathway of imidacloprid degradation by insect gut microbiota.

Resistance to pesticides in insect pest species is increased *via* the metabolism pathway and regulation of various genes and enzymes and is considered a significant problem throughout the globe (Kalsi and Palli, 2017). A study was carried out to examine the resistance of imidacloprid through the regulation of genes in vinegar flies (*Drosophila melanogaster*) and evaluate how imidacloprid metabolites are generated and affect vinegar flies. These questions have been addressed by coupling the genetic tools of gene overexpression and CRISPR gene knock-out with the mass spectrometric technique, the Twin-Ion Method. In this study, *the Cyp6g1* gene, responsible for developing resistance against different insecticides, including imidacloprid, was identified. It found that gut microbes living in vinegar flies were responsible for generating oxidative and nitro-reduced metabolites, which were further interconnected with overexpression of the gene *Cyp6g1.* Additionally, this study revealed that imidacloprid was metabolized into toxic metabolites that were not further degraded into less harmful products and were excreted relatively hardly (Fusetto et al., 2017). In another study, the biodegradation of imidacloprid by the strain *Klebsiella pneumoniae* BCH1 and the effect of its toxic metabolites on silkworm (*Bombyx mori*) were studied. The strain was able to degrade 78% of imidacloprid within a week and produced three metabolites: nitrosoguanidine, imidacloprid guanidine, and 6-chloronicotinic acid by using gas chromatography and mass spectrometry. The toxicity of imidacloprid and its metabolites revealed that they enhanced oxidative stress, lipid peroxidation, protein oxidation, DNA damage, and changed the activity of antioxidant enzymatic status (Phugare et al., 2013; Figure 6).

## Conclusion and future perspectives

The applications of synthetic pesticides are heavily applied throughout the globe to develop insect pests’ infestation strategies. Their toxic residues are accumulated in the agroecosystem, which causes severe threats to animals, humans, birds, and other non-target organisms. Investigating microbial species and their associated enzymes in insect pests’ digestive tracts is essential in agricultural research. Recent advances in independent culture methods such as next-generation sequencing, BLAST analysis, and 16S rRNA analysis have provided new insights into understanding the extensive range of symbiotic microbial communities and their functions with insects. Symbiotic microbial species are very beneficial for the regulation of insect physiology and contribute to a very significant role, such as in insect fitness, by providing amino acids, vitamins, lactic acids, and sterols, enhanced immunity system, food digestion, excretion of waste fluids, host fertility, increased resistance to toxins and external pathogens; and degradation of pesticides and allelochemicals into less toxic products by the production of different hydrolytic enzymes. The biodegradation of pesticides by isolating indigenous insect symbiotic microbial species and their associated catabolic enzymes, genes, and proteins has become an excellent option to clean up the contaminated environment.

Additionally, insects can swiftly obtain novel metabolic activities and colonize new ecological niches through symbiotic interactions with microbiota that have previously fully developed well-tuned metabolic pathways and converted toxic compounds into derivatives. However, the various characteristics of symbiotic microbial species and their associated enzymes that work mutually with insect guts as a superior biocontrol agent are yet to be ascertained. Several omic approaches to predict hidden microbial communities, database approaches for their identification, and systematic biomolecular tools are urgently required to discover unknown features of insect gut microbial species. In the coming days, the relevant discoveries will immensely provide new myriads to explore the proliferation and diversification of insect gut microbial communities and, on the other hand, develop several industrial applications and environmentally friendly technologies for generating wealth, such as the production of biofuels.

## Author contributions

YL conceived the project and contributed to revise the manuscript. SJ prepared the original draft. SA prepared the tables and figures and revised all the manuscript. All authors contributed to the article and approved the submitted version.
